# Prohibitin is overexpressed in Huh-7-HCV and Huh-7.5-HCV cells harboring in vitro transcribed full-length hepatitis C virus RNA

**DOI:** 10.1186/1743-422X-8-424

**Published:** 2011-09-06

**Authors:** Shuang-Suo Dang, Ming-Zhu Sun, E Yang, Meng Xun, Li Ma, Zhan-Sheng Jia, Wen-Jun Wang, Xiao-Li Jia

**Affiliations:** 1 Department of Infectious Diseases, the Second Affiliated Hospital of Medical School of Xi'an Jiao Tong University, Xi'an, Shannxi 710004, P.R. China; 2 Department of the Immunology and Pathogen Biology, the Medical School of Xi'an Jiao Tong University, Xi'an, Shannxi 710061, P.R. China; 3 Center of Diagnosis and Treatment for Infectious Diseases of Chinese PLA, Tangdu Hospital, Fourth Military Medical University, Xi'an, Shannxi 710038, P.R. China

**Keywords:** prohibitin, HCVcc, mRNA level, protein level

## Abstract

**Background:**

Currently, up-regulated proteins and apoptosis in hepatitis C is a hot topic in exploring the pathogenic mechanism of Heptitis C Virus(HCV). Some recent studies shows that prohibitin is overexpressed in cells expressing HCV core proteins, and up-regulated prohibitin is also found in human hepatoma cell line HCC-M, lung cancer, prostate cancer, and other cancers. Prohibitin is an important member of the membrane protein superfamily, and it plays a role of molecular chaperones in mitochondrial protein stability. Meanwhile, it has a permissive action on tumor growth or acts as an oncosuppressor. Based on our previously established the *in vitro *HCV cell-culture system (HCVcc), here we aimed to investigate the different expression profiles of prohibitin in Huh-7-HCV and Huh-7.5-HCV cells

**Methods:**

The total cellular RNA of Huh-7, Huh-7.5, Huh-7-HCV and Huh-7.5-HCV cells were extracted, and then the first-strand cDNA was reversely transcribed. The expression of prohibitin at the mRNA level was assessed by real-time PCR with GAPDH as the control. Furthermore, the expression of prohibitin at the protein level was evaluated by western blot with GAPDH as an internal control.

**Results:**

Our results of real-time PCR showed that the mRNA expression level of prohibitin in Huh-7-HCV cells was 2.09 times higher than that in Huh-7 cells, while, the mRNA level of prohibitin in Huh-7.5-HCV cells was 2.25 times higher than that in Huh-7.5 cells. The results of western blot showed that the protein expression level of prohibitin in Huh-7-HCV cells was 2.38 times higher than that in Huh-7 cells, while the protein expression of prohibitin in Huh-7.5-HCV cells was 2.29 times higher than that in Huh-7.5 cells.

**Conclusions:**

The expression of prohibitin was relatively high in Huh-7-HCV and Huh-7.5-HCV cells harboring *in vitro *transcribed full-length HCV RNA.

## Background

Hepatitis C virus (HCV) is a causative agent of human hepatitis C [1]. HCV infection has become a global health problem with a prevalence of 170 million people, as estimated by the World Health Organization [2,3]. Most (70-80%) HCV infections persist, and about 30% of individuals with persistent infection develop to chronic liver disease, including liver steatosis, cirrhosis and eventually hepatocellular carcinoma [4,5]. However, the response rate is lower than 55% with the current pegylated interferon and ribavirin combination therapy in hepatitis C patients, and HCV also produces complications, such as depression and thyroid dysfunction [6]. Therefore, almost half of patients are not satisfactorily resolved. Moreover, there are no commercial vaccines for hepatitis C prevention, which causes a very difficult problem.

The molecular mechanism of HCV replication, viral persistence and pathogenesis has not yet been fully elucidated. Up to now, the development of specific antiviral therapies and an effective vaccine has been hampered due to the lack of a convenient small animal model. The appearance of full length HCV RNA *in vitro *culture system makes it possible.

Prohibitin is a highly conserved protein, and it is widely distributed in bacteria, plants, fungi, protozoa, and mammals [7,8]. It is a multifunctional protein that localizes at different intracellular sites. Prohibitin is an important member of the membrane protein superfamily, and it mainly exists in the mitochondrial inner membrane. This protein, plays a role of molecular chaperones in maintaining mitochondrial protein stability. It presents in the nucleus, involved in regulation of transcription [9]. In addition, it can be found in the plasma membrane and cytoplasm [10]. In recent years, over-expression of prohibitin has been detected in some tumor cells, including lung cancer, prostate cancer [11], cervical cancer [12], bladder cancer, gastric cancer [13], and breast cancer. Moreover, prohibitin may exert different functional roles, it has a permissive action on tumor growth or acts as an oncosuppressor [14].

In the present study, we compared the expression level of prohibitin in Huh-7 cells harboring full-length HCV RNA (Huh-7-HCV) and control Huh-7 cells. In addition, we evaluated its expression in Huh-7.5-HCV cells and control Huh-7.5 cells. We investigated the prohibitin expression at the mRNA level by real-time PCR and at the protein level by western blot. Our data provided a basis for understanding the function of prohibitin and the HCV pathogenesis which may lead to alternative ways for the treatment of Hepatitis C.

## Materials and methods

### Cell culture and transfection

Huh-7 and Huh-7.5 cells were maintained in Dulbecco's modified Eagle's medium (DMEM) containing 10% heat-inactivated fetal bovine serum (FBS), 0.1 mM nonessential amino acids and 1 × penicillin-streptomycin-glutamine. Plasmid pFL-J6/JFH, containing a chimeric full-length HCV genome, was kindly provided by Professor Rice from Rockefeller University(USA), and it was transcribed to HCV RNA *in vitro*. Subsequently, HCV RNA was electroporately transfected into Huh-7 and Huh-7.5 cells. The *in vitro *HCV cell-culture system (HCVcc) was successfully established. Huh-7-HCV and Huh-7.5-HCV cells were maintained under the same condition as Huh-7 and Huh-7.5 cells. Huh-7, Huh-7.5, Huh-7-HCV and Huh-7.5-HCV cells were cultured in cell incubator at 37°C supplemented with 5% CO_2_. During the cell culture, the supernatant of cell culture was collected at 24, 48 and 72 hours post-electroporation in order to determine the HCV copy numbers. In addition, transmission electron microscopy was used to observe the morphological characteristics of virus particles in transfected Huh-7 and Huh-7.5 cells. At approximately 72 hours post-transfection, Huh-7-HCV and Huh-7.5-HCV cells were washed three times with 1 × phosphate-buffered saline (PBS) and then harvested, whereas Huh-7 and Huh-7.5 cells were collected as control.

### Total RNA isolation, cDNA synthesis, RT-PCR and real-time PCR

Total RNA was extracted from Huh-7, Huh-7.5, Huh-7-HCV and Huh-7.5-HCV cells by TRIzol reagent (Invitrogen, America) according to the manufacturer's instructions, respectively. Briefly, every 5 × 10^6 ^cells were homogenized within 1 mL TRIzol under an RNase free environment. Chloroform was then added (200 μL for each 1 mL TRIzol), and the sample was centrifuged at 12,000 rpm/min at 4°C for 15 min. Subsequently, the aqueous layer was transferred into a new tube, RNA was precipitated with isopropanol, and the precipitate was washed with ethanol. The RNA precipitate was then dissolved in 15 μL DEPC water and analyzed for quantity and quality by a spectrophotometer(CECIL, UK). The integrity of total RNA was examined by electrophoresis on a 0.8% agarose gel, and the quantity was determined based on the absorbance at 260 nm (A260). Finally, the RNA purity was analyzed by the ratio of OD 260/280, and the sample with a value between 1.7 and 2.0 was selected (Table 1).

**Table 1:** Summary of RNA quantification

Cells	Absorbance(260.0 nm)	OD260/OD280	Conc (μg/mL)
Huh7	2.980	1.992	870.4

Huh7.5	2.940	1.976	906.0

Huh7-HCV	2.940	1.912	816.0

Huh7.5-HCV	2.990	1.972	834.0

A two-step reverse transcription PCR was performed. The first-strand cDNA was synthesized from 1 μg of total RNA with AMV Reverse Transcriptase(TAKARA, Japan). To create the real-time PCR standard, prohibitin and Glyceraldehyde 3-phosphate dehydrogenase (GAPDH) were amplified by the specific primers. PCR was performed on MJ Research PTC-200 (MJ Research, America) in a 25 μL reaction system containing the following components: 12.5 μL 2 × Taq PCR Master Mix (Qiagen, Germany), 1 μL cDNA template, 1.5 μL forward primer (10 μM), 1.5 μL reverse primer (10 μM) and 8.5 μL ddH_2_O. Table 2 shows all the primer sequences. Briefly, following a denaturation at 95°C for 5 min, PCR was carried out with 35 cycles at a melting temperature of 95°C for 30 sec, an annealing temperature of 60°C for 30 sec, and an extension temperature of 72°C for 1 min. Finally, an elongation step at 72°C for 30 min was preformed. The amplicons were examined on 1% agarose gel by electrophoresis.

**Table 2:** Primers used for RT-PCR and real-time PCR

Name	Forward primer (5'-3')	Reverse primer (5'-3')
Prohibitin (RT-PCR, 835 bp)	GAAGATCTATGGCTGCCAAAGTGTTTGAG	CGGGATCCTCACTGGGGCAGCTGGA

Prohibitin (real-time PCR, 129 bp)	AAACAGGTGGCTCAGCAGGAA	CAGTGAGTTGGCAATCAGCTCAG

GAPDH (real-time PCR, 138 bp)	GCACCGTCAAGGCTGAGAAC	TGGTGAAGACGCCAGTGGA

In order to examine the mRNA expression of prohibitin, the expression of prohibitin and GAPDH genes was quantified by real-time PCR, and GAPDH served as an internal control. The length of the amplified PCR products ranged from 50 to 150 bp as recommended by Applied Biosystems. A total of 20 ng cDNA was used as template in the reaction. All real-time PCR assays were performed in triplicate with Bio-Rad iQ5 Multicolor Real-time PCR Detection System (America)according to the manufacture's protocol. Briefly, following a denaturation at 95°C for 5 sec, real-time PCR was carried out with 50 cycles at a melting temperature of 95°C for 30 sec, an annealing temperature of 60°C for 30 sec, and an extension temperature of 72°C for 10 sec. Data analysis was performed with the Sequence Detector System software. The relative quantification was calculated by the ^ΔΔ^Ct method with GAPDH as the housekeeping gene and the non-transfected cells as the baseline, and the results were expressed as fold-change.

### Protein extraction, SDS-PAGE and western blotting

To compare protein expression between the transfected and non-transfected cells, the total proteins were prepared by RIPA cell lysate. Proteins of interest were separated by SDS-polyacrylamide gel electrophoresis (PAGE) with a 10% polyacrylamide gel, and the protein concentration loaded on the SDS-PAGE was always at 1 mg/mL. Proteins were transferred to nitrocellulose membranes and then detected by western blotting under the recommended conditions. Rabbit anti-human IgG (Santa, America) was used as the primary antibody, goat anti-rabbit IgG conjugated with horseradish peroxidase (HRP) was used as the secondary antibody, and GAPDH was used as control. The antigen-antibody complex was detected by an enhanced chemiluminescence (ECL) kit following the manufacturer's protocol. The chemiluminescent signal of each band was analyzed by gel image analysis system. The expression levels of prohibitin and GAPDH in Huh-7-HCV and Huh-7.5-HCV cells were compared with those in Huh-7 and Huh-7.5 cells separately.

## Results

### Detection of HCV at the RNA level in infected cellular supernatant and transmission electron microscopy observation

We detected HCV RNA in the supernatant of infected Huh-7 and Huh-7.5 cells. It reached a peak value at 48 hours post-transfection (6.4 × 10^6 ^copies), and the HCV RNA maintained at the same level in the supernatant until 72 hours post-transfection. The electron microscopy demonstrated that the cytoplasm of Huh-7-HCV and Huh-7.5-HCV cells contained a large number of spherical structures, which might be the nucleocapsid-like particles. Some characteristics of Flaviviridae virus infection appeared in the internal cellular structure, the rough endoplasmic reticulum increased, the endoplasmic network management dilated, vacuolar structures appeared in the cytoplasm, and a large number of HCV nucleocapsid-like particles of inclusion body presented in Huh-7-HCV and Huh-7.5-HCV cells. We did not find any form of virus-like particles in the control cells. Moreover, we did not observe the hyperplasia, vacuolar membrane structure and formation of inclusion bodies in the control cells.

### Prohibitin expression at the mRNA level was up-regulated in Huh-7-HCV and Huh-7.5-HCV cells by real-time PCR

**Figure 1:**
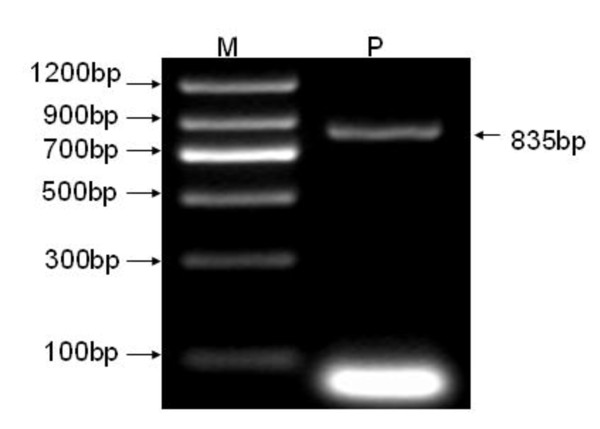
**Prohibitin was amplified by RT-PCR**. Note: M: DNA Marker || (Qiagen, Germany); P: Prohibitin.

**Figure 2:**
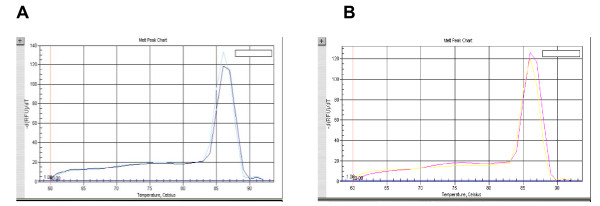
**Melt curve of prohibitin expression detected by real-time PCR**. A. Melt curve of prohibitin in Huh-7 and Huh-7-HCV cells. B. Melt curve of prohibitin in Huh-7.5 and Huh-7.5-HCV cells.

**Figure 3:**
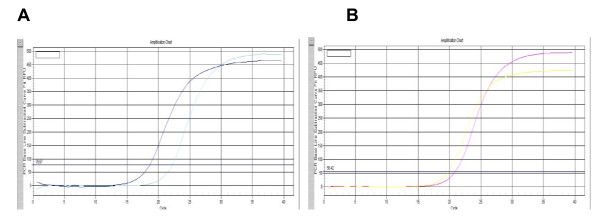
**The amplification curve of prohibitin expression detected by real-time PCR**. A. The amplification curve of prohibitin in Huh-7 and Huh-7-HCV cells. B. The amplification curve of prohibitin in Huh-7.5 and Huh-7.5-HCV cells.

**Figure 4:**
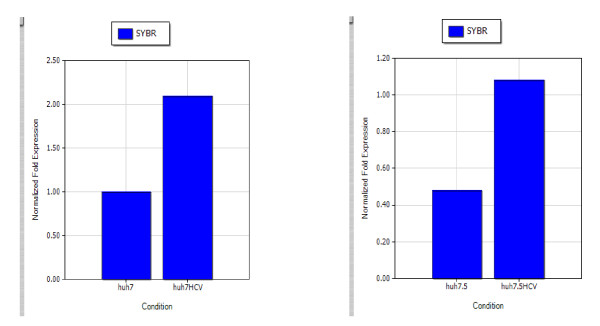
**Prohibitin mRNA relative expression rate in either Huh-7 and Huh-7-HCV cells, or Huh-7.5 and Huh-7.5-HCV cells**.

The extracted total cellular RNA was examined by electrophoresis on a 0.8% non-denaturing agarose gel. Figure 1 shows that a 835-bp fragment of the full coding sequence of prohibitin was successfully amplified by RT-PCR without unspecific amplification. Figure 2 shows the melt curve of prohibitin expression. A Single peak was detected in all amplifications, suggesting that the primers were properly designed and the prohibitin was specifically amplified. Figure 3 shows the amplification curve of prohibitin. Figure 4 shows that prohibitin expression in Huh-7-HCV cells was 2.09 times higher than that in Huh-7 cells, and prohibitin expression in Huh-7.5-HCV cells was 2.25 times higher than that in Huh-7.5 cells. The expression level of prohibitin was significantly higher expressed in Huh-7 and Huh-7.5 cells transfected with full-length HCV RNA.

### Prohibitin expression at the protein level was up-regulated in Huh-7-HCV and Huh-7.5-HCV cells by western blot analysis

**Figure 5:**
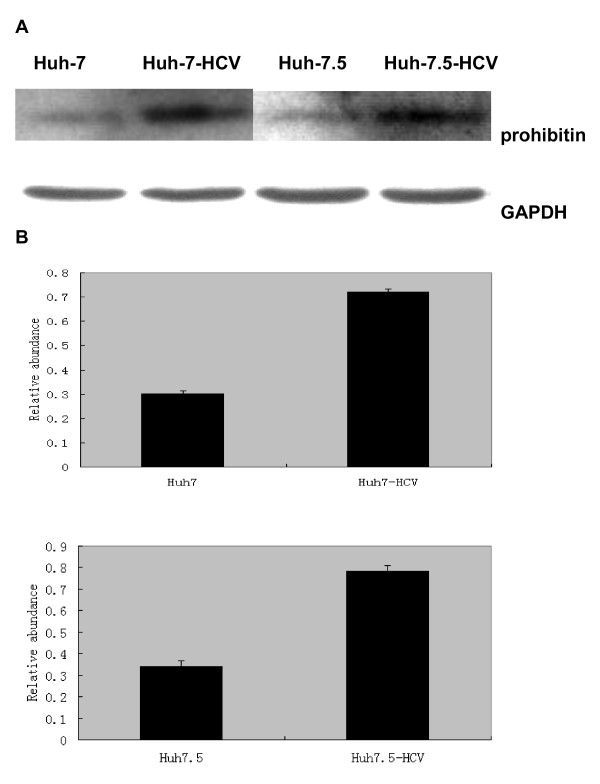
**The prohibitin expression at the protein level by western blot**. A. Prohibitin expression in Huh-7, Huh-7-HCV, Huh-7.5 and Huh-7.5-HCV cells. B. Relative prohibitin expression normalized to GAPDH.

Using western blotting, we compared the chemiluminescent signals of prohibitin and GAPDH in Huh-7-HCV and Huh-7.5-HCV cells with those in Huh-7 and Huh-7.5 cells, and the ratio between prohibitin and GAPDH reflected changes of prohibitin. The results showed that the ratio of prohibitin/GAPDH was 0.721 ± 0.025 and 0.30 ± 0.012 in Huh-7-HCV cells and Huh-7 cells, respectively, demonstrating that the prohibitin expression in Huh-7-HCV cells was significantly higher than that in Huh-7 cells. Furthermore, the ratio of prohibitin/GAPDH was 0.78 ± 0.070 in Huh-7.5-HCV cells, which was significantly higher than that in Huh-7.5 cells (0.34 ± 0.025) (Figure 5). The statistical analysis revealed that the difference between these two groups was statistically significant (P < 0.05). Therefore, we confirmed that the expression level of prohibitin was increased in Huh-7-HCV and Huh-7.5-HCV cells. These findings demonstrated that the over-expression of prohibitin was related to HCV infection.

## Discussion

Currently, much attention has been paid to the relationship between the up-regulated proteins after HCV infection and the pathology of liver injury. It is known that HCV creates the living environment for its reproduction, and it survives in the host cells by affecting the host cell apoptosis. Pathological changes in the liver tissue of hepatitis C patients include the lobular necrosis, the portal area like edge of eosinophilic necrosis, acidophilic corpuscle or Councilman bodies. Furthermore, apoptosis plays an important role in the pathogenesis of hepatitis C.

We hypothesized that the up-regulated proteins by HCV infection had a direct connection with the pathological liver injury. The new discovery of up-regulated proteins and elucidation of pathogenic mechanisms could provide some important insights into the treatment of hepatitis C. Tsutsumi et al. (2009) reported that the prohibitin expression is up-regulated not only in the HCV cells expressing core-protein, but also in the whole genome replication cells as well as the liver of core-gene transgenic mice [15]. Their results suggest that high expression of prohibitin is significantly related to the cellular mitochondrial metabolism.

McClung et al. first cloned mammalian prohibitin gene in 1989. The prohibitin gene is located in chromosome 17q21, encoding a protein with a molecular weight of 32 kD [16]. It is known that prohibitin protein functions as molecular chaperons and is involved in mitochondrial protein stability [17]. In addition, prohibitin protein may contribute to the regulation of cell cycle [18], regulation of cell signal transduction, inhibition of cell proliferation [19], induction of apoptosis [20], anti-aging, maintenance of cell homeostasis and many cellular activities of life. However, its functional roles have not been well-defined and its specific mechanisms are unknown.

Jang et al. found that the expression of prohibitin is down-regulated in gastric cancer [21]. Gamble et al. found that the expression of prohibitin is down-regulated more than 50% in androgen-mediated prostate cancer [22]. In recent years, with the advance of experimental techniques, more and more studies show that the expression of prohibitin is up-regulated in tumor tissues and cells. For example, Seow et al. reported that the expression of prohibitin is up-regulated when detecting differentially expressed proteins in human hepatoma cell line HCC-M using two-dimensional electrophoresis [23].

In our study, we found that prohibitin was up-regulated in human hepatoma cells transfected with full-length HCV RNA compared with the control cells. Real-time PCR revealed that the prohibitin expression at the mRNA level in Huh-7-HCV cells was 2.09 times higher than that in Huh-7 control cells; while the prohibitin expression at the mRNA level in Huh-7.5-HCV cells was 2.25 times higher than that in Huh-7.5 control cells. Western blot analysis demonstrated that the prohibitin expression at the protein level in Huh-7-HCV cells was 2.38 times higher than that in Huh-7 control cells; while the prohibitin expression at the protein level in Huh-7.5-HCV cells was 2.29 times higher than that in Huh-7.5 control cells. Prohibitin was over-expressed both in Huh-7-HCV and Huh-7.5-HCV cells, which confirmed the universility of prohibitin up-regulating. Overexpression of prohibitin may have a close relationship with HCV RNA transcription, its down-regulation might decrease the replication of HCV RNA. In future, we will investigate the biological role of prohibitin and reveal the relationship between prohibitin and apoptosis. Those studies will provide a better understanding on the pathogenic mechanism of HCV and develop better approaches for the treatment of Hepatitis C.

## Competing interests

The authors declare that they have no competing interests.

## Authors' contributions

DSS supervised all phases of the project: conception and design of the experiments, analysis of the results and writing of the manuscript. SMZ performed the experiments, analysed the results, and drafted the manuscript. YE and XM designed the study. ML, JZS, WWJ, JXL analyzed the results. All authors read and approved the final manuscript.
